# Failure Tolerance of Motif Structure in Biological Networks

**DOI:** 10.1371/journal.pone.0020512

**Published:** 2011-05-26

**Authors:** Baharan Mirzasoleiman, Mahdi Jalili

**Affiliations:** Department of Computer Engineering, Sharif University of Technology, Tehran, Iran; University of Maribor, Slovenia

## Abstract

Complex networks serve as generic models for many biological systems that have been shown to share a number of common structural properties such as power-law degree distribution and small-worldness. Real-world networks are composed of building blocks called motifs that are indeed specific subgraphs of (usually) small number of nodes. Network motifs are important in the functionality of complex networks, and the role of some motifs such as feed-forward loop in many biological networks has been heavily studied. On the other hand, many biological networks have shown some degrees of robustness in terms of their efficiency and connectedness against failures in their components. In this paper we investigated how random and systematic failures in the edges of biological networks influenced their motif structure. We considered two biological networks, namely, protein structure network and human brain functional network. Furthermore, we considered random failures as well as systematic failures based on different strategies for choosing candidate edges for removal. Failure in the edges tipping to high degree nodes had the most destructive role in the motif structure of the networks by decreasing their significance level, while removing edges that were connected to nodes with high values of betweenness centrality had the least effect on the significance profiles. In some cases, the latter caused increase in the significance levels of the motifs.

## Introduction

Many real-world complex systems can be described as networks. Examples include the Internet, World Wide Web, the brain functional/anatomical networks, genetic regulatory networks, metabolism of biological species, ecological systems, and networks of author collaborations [1], [2], [3]. Scholars have found that many real-world networks from physics to biology, engineering and sociology have some common structural properties such as power-law degree distribution [4] and small-worldness [5]. Studying the properties of such networks could shed light on understanding the underlying phenomena or developing new insights into the system. For example, studying biological networks helps us to better understand the organization and evolution of their units [6]. Recent developments in computing facilities let researchers mine the data of real-world networks to discover their topological properties.

In its simplest form, a network consists of a set of discrete elements called nodes (or vertices), and a set of connections linking these elements called edges (or links). One of the tricky parts of research in this field is to extract the graph of system under study that is to identify the individual nodes and reconstruct the links connecting them. As network structure is identified, its structural and dynamical properties are investigated. Network motifs are among such attributes that are usually tested for natural networks. It has been shown that networks in various fields exhibit interesting features in terms of repeated occurrences of certain subgraphs, i.e. motifs [7], [8]. Network motifs are patterns (particular subgraphs) that statistically overrepresented or underrepresented within the network. The significance of a particular subgraph in a network is usually measured by comparing its occurrences in the original network against some properly randomized networks. Network motifs have been identified in networks from different branches of science and are suggested to be the basic building blocks of most complex networks [9]. Analysis of this over/under abundant substructures can help us in determining different network properties and functions such as its hierarchal structure. The motif structure of a network might be important in determining its dynamical properties. For example, the evolution of cooperativity [10], [11], has been linked to the motif structure in real networks [12].

One of the important features of many engineering and biological networks is robustness against component failure [13], [14]. Real-world networks may undergo random or systematic failures and consequently lose some of their components, i.e. nodes and/or edges. Therefore, it is essential to investigate the tolerance of critical network properties to errors– failures of randomly chosen nodes and/or edges of the networks and attacks– systematic failures of components that play a critical role in the network [15], [16]. It has been shown that many biological networks exhibit high degrees of robustness against random errors that might happen in their structure [13], [14], [15], [17], [18]. In general, it has been shown that scale-free networks, i.e. networks whose node-degree distribution follows a power-law, are robust against errors, but, at the same time, they are fragile in response to systematic attacks [15], [19], [20], [21]. Several measures have been proposed for measuring robustness of networks against attacks and errors. One of the frequently used ones is the largest connected component whose size scales linearly with the number of nodes in the network [15], [20], [22]. Efficiency is another important measure that is studied in the context of robustness of complex networks against attacks/errors [19]. The errors/attacks influence the evolution of dynamical processes happening on the networks. Network cooperativity, for instance, has been shown to be extremely robust against random failures, while it is fragile when nodes with maximum degree are removed from the network [23].

In this paper we investigated the influence of link failures in the profile of network motifs. We considered protein structure network [8] and functional network of human brain extracted through functional magnetic resonance imaging technique [24]. A number of strategies for choosing candidates edge for removal were taken into account that included random removal, removing edges based on the degrees of the end nodes, based on the betweenness centrality of the nodes, and based on the closeness centrality of the nodes. We then compared the profile of the network motifs as a function of the percentage of removed edges. Interestingly, different failure strategies resulted in different pattern of changes in the motif structure where the strategy based on the betweenness centrality was the most different with the other three.

## Materials and Methods

### Motif Structure

Many real-world complex networks have been shown to be composed of well-defined building blocks called *motifs*. Network motifs are patterns of interconnection or subgraphs that occur in natural networks much more frequent than those in randomized networks [7], [8]. They can be thought of as simple building blocks of complex networks [8], which can provide valuable information about structural design principles of networks. First discovered in the gene regulation (transcription) network of the bacteria *Escherichia coli* by Alon and his team [8], [25], they have been found in many networks ranging from biochemistry to neurobiology networks, ecology, and engineering [9], [26], [27]. Study of network motifs is therefore propitious for revealing the basic building blocks of most complex networks.

Some studies have related the function of networks to the structure of their motifs. Transcription networks are among those heavily studied both theoretically and experimentally. For example, negative-autoregulation which is one of the simplest and most abundant motifs in *Escherichia coli* has been shown to be response-acceleration and repair system [28]. Positive-autoregulation motif is important in biomodal distribution of protein levels in cell population [29]. Feed-forward loop that is commonly found in many gene systems and organisms is important in speeding up the response time of the target gene expression following stimulus steps, pulse generation and cooperativity [30]. Dense Overlapping Regulons that occur when several regulators combinatorially control a set of genes with diverse regulatory combinations, has also been shown to be important in the function of *Escherichia coli* [31].

Although subgraphs of different sizes can be studied in natural networks, among them, biological networks contain three and four-node substructures far more often compared to randomized networks with similar structural properties. Many beneficial outcomes have been ensued from these observations. Often the network motifs are detected by comparing the network against a null hypothesis, that is, the number of appearance of a specific subgraph is counted in the networks and is subsequently compared with the number of appearances in properly randomized networks. The randomized networks can be constructed in various ways. However, they should at least share some common properties with the original network. For example, the randomized networks should have the same number of nodes and edges with the original network. One possible method is to build the corresponding Erdos-Renyi version for the networks [32]. A better way of constructing the randomized networks is to preserve not only their size and average degree but also their degree distribution or at least degree sequence. This can be simply done by shuffling the adjacency matrix [33]. Many of the motif detection strategies use this algorithm for constructing the randomized version of the original network under study. The motif detection algorithm can be summarized as follows [7], [8]:

1. Consider a specific subgraph *i*
2. Count the number of appearances of the subgraph *i* in the network *N_i_*
3. Generate sufficiently large number of randomized networks with the same number of nodes and degree distribution as the original network
4. Count the number of appearances of the subgraph *i* in each of the randomized networks
5. Compute the average number of appearances of the subgraph *i* in the randomized networks <*N*rand*_i_*> and its standard deviation std(*N*rand*_i_*)
6. Compute the significance of appearances of the subgraph *i* as
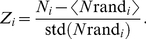
(1)
7. The networks motifs are subgraphs for which the probability *P* of appearing in the randomized networks an equal or greater number of times than in the original network is lower than a cutoff value (e.g. *P*<0.01). Thus, higher absolute values of *Z*-scores correspond to more significant network motifs.

Note that the Z-score of a motif can be positive or negative; positive when it is highly overrepresented in the original network as compared to randomized ones and negative when it is highly underrepresented.

It has also been proposed to normalize the *Z*-scores [7]. The Z-score of an specific motif may depend on the network size and it tends to be higher in larger networks [7]. Since complex networks may vary widely in size, one can take an approach that enables to compare different network's local structure. To this end, the normalized *Z*-scores can be calculated as
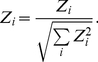
(2)


The normalization emphasizes the relative significance of subgraphs rather than the absolute significance, which is important for comparison of subgraph of different sizes [7].

A motif of size *k* is called a *k*-motif. The runtime of counting process grows very fast with *k*. This is one of the reasons why only small *k*-motifs (usually three- or four-nodes) have been studied in most of the works. Different tools have been developed for the detection and analysis of network motifs such as Mfinder [34], MAVisto [35], and FANMOD [36]. In this work we used Mfinder, which uses a semi-dynamic programming algorithm in order to reduce the running time [34]. It also uses an efficient sampling algorithm that significantly reduces the running time compared to the cases where all edges are visited.

### Two Biological Networks

Techniques from complex networks have been widely applied to many biological systems (e.g. see reviews [6], [13], [37]). Recent developments in designing efficient techniques in molecular biology have led to extraordinary amount of data on key cellular networks in a variety of simple organisms [8], [38], [39], [40], [41]. This allowed scholars to study networks such as protein interaction, transcriptional regulatory, and metabolic in different organisms. Networks have also been widely studied in neurosciences [42], [43], [44]. The brain networks can be studied on a micro-scale containing a number of neurons with some excitatory/inhibitory connections in-between [45], [46], [47]. However, this approach cannot be used for studying the whole-brain connectivity network. For such cases, one should use functional magnetic resonance imaging, diffusion imaging, magnetocephalography, or electroencephalography techniques to extract the large-scale functional/anatomical brain connectivity networks [48], [49], [50], [51].

**Figure 1:**
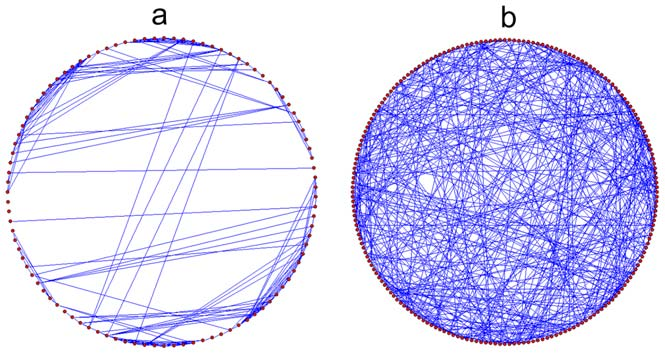
Topology of sample biological networks. (a) Protein structure network [7] and (b) human brain functional network extracted through functional magnetic resonance imaging [24].

In this work, we have considered two biological networks: protein structure network [7], and human brain functional network extracted through functional magnetic resonance imaging [24]. Figure 1 shows their structure by representing the nodes and edges connecting them. Their properties including, size, average degree, standard deviation of the degrees, average path length and clustering coefficient is represented in Table 1. We used Mfinder to determine the significance of all three- and four-nodes subgraphs of these networks. In order to obtain a high level of accuracy, we set the parameters of random network generation algorithm and counting motifs in the tool as follows [34]

- Number of random networks = 10000
- Uniqueness threshold is ignored
- No threshold on mfactor to use when counting motifs
- No threshold on Z-score to use when counting motifs
- Default values were considered for other parameters, including switching method for generating random networks.

**Table 1:** Characteristics of considered biological networks.

Network Type	N	<k>	std(k)	P	C
Protein structure	99	4.2828	0.4748	5.2607	0.3600
Functional human brain	200	4.5400	0.5690	5.2200	0.2858

First columns: the name of the networks. Second to sixth columns: network size (N), average node-degree (<k>), standard deviation of node-degree (std(k)), average characteristic path length (P), and clustering coefficient (C).

Table 2 summarizes the set of three- and four-node motifs with their corresponding normalized and non-normalized Z-scores in the networks. As we can see motif #7 — a four-node motif with five edges — has the highest positive Z-score, and thus, is the most significance motif structure in both of the networks and can be considered as the dominant motif. On the other hand, motif#1 has the highest negative Z-score in both of the networks, and thus, is the most significant anti-motif in the set of three- and four-node subgraphs. There is a significant direct correlation between the Z-scores of the motifs in these two networks (*r* = 0.9328, *P*<0.001; Pearson linear correlation and *r* = 0.9286, *P*<0.0025; Spearman rank correlation). This indicates the similarity of these two networks in the structure of their building blocks, i.e. #2, #5, #7, and #8, have always positive Z-score, i.e. they are significantly more abundant in these networks as compared to random networks. As the clustering coefficient of the real networks is relatively large (see Table 1), it seems natural that the subgraphs that include a triangle structure have a positive Z-score. In some sense, the Z-score of motifs #5, #7 and #8 seems strongly dependent on the Z-score of motif #2. The negative Z-score of motif #1 seems also correlated to the positive Z-score of motif #2. Subgraph #1 and #4 (motif #6 that has small Z-score and is not a significant motif) has always negative Z-score meaning that they are anti-motifs appearing much less in the original networks as compared to random ones.

**Table 2:** Significance profiles of motifs in the networks.

Network Type		Protein structure			Functional Human brain		
MotifNumber	MotifStructure	Motif frequencies	Non-normalizedZ-scores	normalizedZ-scores	Motif frequencies	Non-normalizedZ-scores	normalizedZ-scores
#1		544	−29.581	−0.0060	1388	−44.913	−0.0034
#2		130	25.086	0.0051	187	38.600	0.0029
#3		294	−20.086	−0.0041	1008	−33.844	−0.0025
#4		1359	−21.871	−0.0044	4020	−34.167	−0.0026
#5		661	11.529	0.0023	1196	24.000	0.0018
#6		29	−1.687	−0.0003	88	6.351	0.0005
#7		150	37.333	0.0076	205	81.360	0.0061
#8		38	31.666	0.0064	19	17.272	0.0013

All possible three- and four-node motifs along with the corresponding frequency of occurrence, normalized and non-normalized Z-scores in the networks.

### Random and Systematic Failures in the Edges

Random or systematic failures can occur in some of the networks' components, i.e. nodes and edges. For example in protein-protein interaction network, while attacking nodes may correspond to breakdown of polypeptides by appropriate enzymes, attacking edges of the network can be interpreted as preventing physical interaction between two polypeptides in order to prevent carrying out their biological functions. In this work we considered failures in the edges and investigated its influence on the profile of the motif structure of the networks. Failures in the networks are of two types, in general: random failures that are called errors or systematic failures that are called attacks.

Let define some preliminary metrics of graph theory. Consider an undirected and unweighted network with adjacency matrix *A* = (*a_ij_*), *i*, *j* = 1, …, *N*, where *N* is the size of the network. Let denote the edges between the node *i* and the node *j* by *e_ij_*. The degree of the node *i* can be obtained as
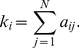
(3)


Edge betweenness centrality (load) is a centrality measure of an edge in a graph, which counts the number of shortest paths passing through the edge. The betweenness centrality *L_ij_* of the edge *e_ij_* between nodes *i* and *j* that is defined by [52]

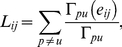
(4)where Γ*_pu_* is the number of shortest paths from nodes *p* to *u* in the graph and Γ*_pu_*(*e_ij_*) is the number of these shortest paths making use of *e_ij_*. The betweenness centrality of an edge is indeed the load of shortest paths using that edge, i.e. the larger the betweenness centrality of an edge is the more its significance in the formation of the shortest paths in the network is.

In a topological space and complex network analysis, closeness is a basic and important concept. In graph theory closeness is the inverse of the sum of the shortest distances between each node in the network. In other worlds, the closeness centrality *C_i_* of node *i* is defined as
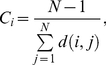
(5)where *d*(*i*,*j*) is the length of the shortest path between the nodes *i* and *j*. Indeed, the closeness centrality of node *i* is the inverse of the average shortest path from *i* to other nodes in the network.

We considered different failure strategies in the networks. In order to choose candidate edges for removal four strategies were considered as follows:

1. Random failure: at each step, one edge was randomly chosen and removed from the network.
2. Systematic failure based on the node degrees: at each step, the quantity *k_i_k_j_* was calculated for each edge *e_ij_*, and then, the edge with the maximum amount of *k_i_k_j_* was removed from the network. If some edges have the same value of *k_i_k_j_*, one of them was removed randomly.
3. Systematic failure based on the edge betweenness centrality: at each step, the quantity *L_ij_* was calculated for each edge *e_ij_*, and then, the edge with the maximum amount of *L_ij_* was removed from the network.
4. Systematic failure based on the node closeness centrality: at each step, the quantity *C_i_C_j_* was calculated for each edge *e_ij_*, and then, the edge with the maximum amount of *C_i_C_j_* was removed from the network.

## Results and Discussion

We applied the failure strategies to the networks, i.e. protein structure and human brain functional networks. Starting from the original network and at each step, a candidate edge (based on a failure strategy) was removed, and the *Z*-scores of all undirected three-and four-nodes subgraphs were calculated for the resulting network. Since in calculating the subgraph ratio profile described by Eq. (2) all terms are affected by the removal, the effect of removal on each subgraph is not clear. Therefore, we studied the non-normalized Z-scores. After each removal, the profiles of non-normalized Z-scores were calculated with respect to corresponding randomized networks with the same degree distribution. Then, the results were displayed as a function of the percentage of removed edges. Because motifs correspond to particular functions, the evolution of the frequencies of the motifs with the percentage of removed edges is at least as important as their Z-score. The Z-scores are indeed relativized to a random network, and thus, from this metric it is not clear how the frequency of each subgraph changes. To understand better what happens with motifs composition in the considered networks and their randomized alternatives, we also plotted the motifs frequencies *vs.* the percentage of removed edges.

**Figure 2:**
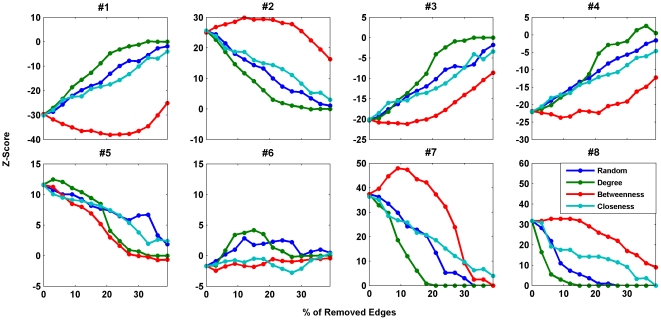
*Z*-score of motifs #1–#8 as a function of the percentage of removed edges for protein structure network. The blue, green, red and cyan lines show the changes in the *Z*-score for random failure (failure strategy 1), systematic failure based on node degrees (failure strategy 2), systematic failure based on betweenness centralities (failure strategy 3), and systematic failure based on node closeness centralities (failure strategy 4), respectively. The case with random failure is averaged over 10 realizations.

**Figure 3:**
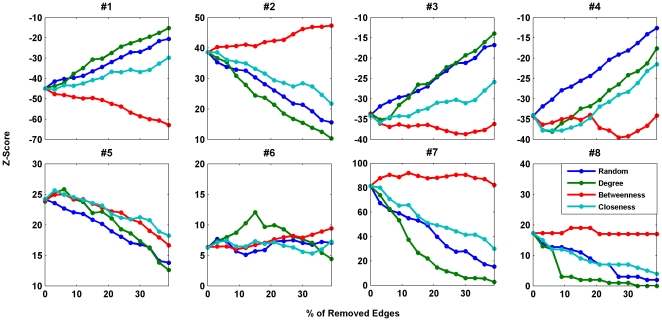
*Z*-score of motifs #1–#8 as a function of the percentage of removed edges for human brain functional network. The blue, green, red and cyan lines show the changes in the *Z*-score for random failure, systematic failure based on node degrees, systematic failure based on betweenness centralities, and systematic failure based on node closeness centralities, respectively. The case with random failure is averaged over 10 realizations.

Figures 2 and 3 show the profile of *Z*-scores of motifs of size three and four in the networks. We removed edges based on different strategies, i.e. random failure (failure strategy 1), systematic failure based on node degrees (failure strategy 2), systematic failure based on betweenness centralities (failure strategy 3), and systematic failure based on node closeness centralities (failure strategy 4). It can be seen that random failure in the edges and systematic failures based on degree or closeness centrality always weakened the significance of the subgraphs in the resulting networks, i.e. the significance level of the *Z*-scores decreased. However, the systematic failure based on the betweenness centralities showed different effects. Removing edges with the highest betweenness centrality resulted in networks with increasing significance of some of their motifs, while the significance of some other motifs decreased.

Interestingly, systematically removing those edges tipping to high degree nodes had the most catastrophic influence in decreasing the absolute value of the *Z*-scores, i.e. decreasing the significance level of the network motifs and anti-motifs, in both networks. In other words, the more the degree of the vertices at the ends of an edge is the more critical that edge is for the motif structure. Network motifs are important in its functionality. For example, the dynamical property of many real-world networks are highly correlated with the relative abundance of motifs in those networks [12], [53], [54]. In gene regulatory networks, their motif structure is important in the response time of the target gene expression following stimulus steps, pulse generation and cooperativity [30]. Thus, the degree-based attack on the edges might affect the networks' functionality through weakening the significance of their motifs. As a result, in order to make the network motifs robust against such attacks, one should protect the edges connecting the hub nodes in the network. On the other hand, preventing the system from doing a well-specific functionally might be desired in some applications. This can be done by removing those edges connecting hub nodes in the network, if such functionality is linked to the motif structure of the network.

Another interesting observation is that, in most cases, random removal of the edges is not the weakest strategy in breaking the significance of the motifs. In some cases, e.g. motif #4 in human brain functional network, it is the most effective strategy in reducing the significance of network motifs. Therefore, in real-world biological networks, such as the two examples studied in this work, errors, i.e. random failures, can be as effective as attacks, i.e. systematic failures, in influencing the motif structure.

Among different strategies for systematic removal of the edges the one based on the betweenness centrality has the least influence on the *Z*-scores. The profiles of Z-scores are largely robust against systematically removing the highly loaded edges. In some cases, e.g. motif #1 and motif #2, removing such edges resulted in increasing the significance level of the motif structure in the final networks. This can be due to the fact that the edges with high betweenness centrality are probably those connecting two parts of the network, i.e. bridges or local bridges. Such links usually participate in few graphlets of size three or four. Removing such edges may increase the relative abundance of the graphlets in the resulting network as compared to those in the randomized networks.

**Figure 4:**
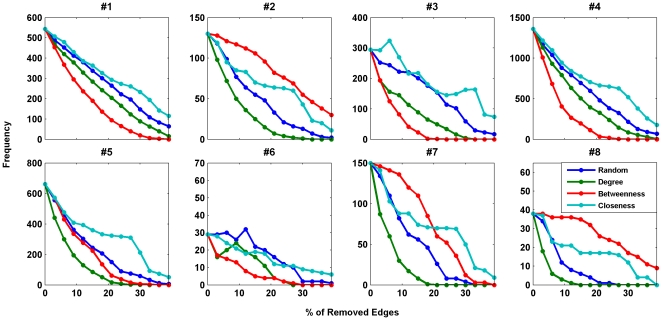
Frequencies of motifs #1–#8 as a function of the percentage of removed edges for protein structure network. The blue, green, red and cyan lines show the changes in the *Z*-score for random failure, systematic failure based on node degrees, systematic failure based on betweenness centralities, and systematic failure based on node closeness centralities, respectively. The case with random failure is averaged over 10 realizations.

**Figure 5:**
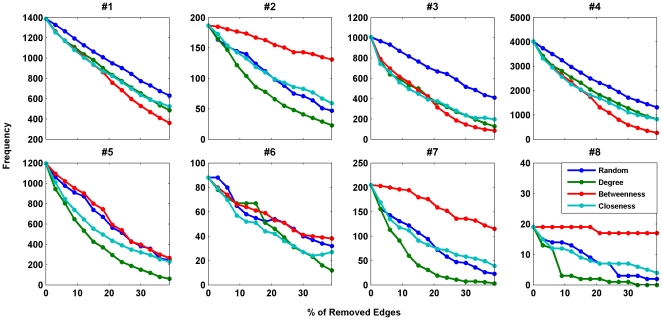
Frequencies of motifs #1–#8 as a function of the percentage of removed edges for human brain functional network. The blue, green, red and cyan lines show the changes in the *Z*-score for random failure, systematic failure based on node degrees, systematic failure based on betweenness centralities, and systematic failure based on node closeness centralities, respectively. The case with random failure is averaged over 10 realizations.

Figures 4 and 5 show the rate of decrease of the motifs' frequencies in different failure strategies. The results revealed that the removal strategy based on the betweenness centrality is the most influential one in decreasing the number of the anti-motifs, i.e. motif #1, motif #3 and motif #4. For subgraphs with positive Z-scores, removing edges connected to high degree nodes in the network had the most influence in decreasing the motifs frequencies. Similar to the case of subgraph significance profiles, random strategy is not the weakest strategy in reducing the number of subgraphs in most cases. It is usually more effective than systematic failures based on betweenness or closeness centrality. Therefore, different failure strategies have different influence on the frequency of occurrence and significance profile of the network motifs in biological networks. Our results showed that removing edges connected to high degree nodes in the network has the most influence, in general, in decreasing the relative appearance of three and four-node subgraphs in the resulting networks as compared to random networks. This strategy also plays an important role in decreasing the motifs frequency. On the other hand, removing the highly loaded edges has the least influence on the changes of the motifs significance profiles.

In summary, we investigated the effect of random and systematic failures on the profile of their three- and four-node motifs. As network examples we considered protein structure network and human brain functional network extracted through functional magnetic resonance imaging. We considered four strategies to choose edges for removal: random failure where the edges are randomly removed, systematic failure in the edges connected to high degree nodes, systematic failure in the edges with high betweenness centrality, and systematic failure in the edges connected to the nodes with high values of closeness centrality. We showed that although biological networks have been shown to be robust against random failures in terms of network connectedness and efficiency, such failures can have destructive effects on network motifs. Degree-based systematic failure had the most destructive role in most cases, i.e. causing in the largest decrease in the frequency of occurrence and absolute value of the *Z*-scores. While, attacks in the highly loaded edges had the least influence on the motif profile, and in some cases, such attacks resulted in networks enhancing the significance of the motif structures. Since motifs play important roles in the functionality of real-world biological networks, these results are important in studying error and attack tolerance of biological networks.
